# Individual Trabeculae Segmentation (ITS)–Based Morphological Analysis of High-Resolution Peripheral Quantitative Computed Tomography Images Detects Abnormal Trabecular Plate and Rod Microarchitecture in Premenopausal Women With Idiopathic Osteoporosis

**DOI:** 10.1002/jbmr.50

**Published:** 2010-01-25

**Authors:** X Sherry Liu, Adi Cohen, Elizabeth Shane, Emily Stein, Halley Rogers, Shannon L Kokolus, Perry T Yin, Donald J McMahon, Joan M Lappe, Robert R Recker, X Edward Guo

**Affiliations:** 1 Bone Bioengineering Laboratory, Department of Biomedical Engineering, Columbia UniversityNew York, NY, USA; 2 Division of Endocrinology, Department of Medicine, Columbia UniversityNew York, NY, USA; 3 Department of Medicine, Osteoporosis Research Center, Creighton UniversityOmaha, NB, USA

**Keywords:** bone microstructure, high-resolution peripheral quantitative computed tomography, individual trabeculae segmentation, trabecular plate/rod

## Abstract

Idiopathic osteoporosis (IOP) in premenopausal women is a poorly understood entity in which otherwise healthy women have low-trauma fracture or very low bone mineral density (BMD). In this study, we applied individual trabeculae segmentation (ITS)–based morphological analysis to high-resolution peripheral quantitative computed tomography (HR-pQCT) images of the distal radius and distal tibia to gain greater insight into skeletal microarchitecture in premenopausal women with IOP. HR-pQCT scans were performed for 26 normal control individuals and 31 women with IOP. A cubic subvolume was extracted from the trabecular bone compartment and subjected to ITS-based analysis. Three Young's moduli and three shear moduli were calculated by micro–finite element (µFE) analysis. ITS-based morphological analysis of HR-pQCT images detected significantly decreased trabecular plate and rod bone volume fraction and number, decreased axial bone volume fraction in the longitudinal axis, increased rod length, and decreased rod-to-rod, plate-to-rod, and plate-to-plate junction densities at the distal radius and distal tibia in women with IOP. However, trabecular plate and rod thickness did not differ. A more rod-like trabecular microstructure was found in the distal radius, but not in the distal tibia. Most ITS measurements contributed significantly to the elastic moduli of trabecular bone independent of bone volume fraction (BV/TV). At a fixed BV/TV, plate-like trabeculae contributed positively to the mechanical properties of trabecular bone. The results suggest that ITS-based morphological analysis of HR-pQCT images is a sensitive and promising clinical tool for the investigation of trabecular bone microstructure in human studies of osteoporosis. © 2010 American Society for Bone and Mineral Research.

## Introduction

Osteoporosis, characterized by low bone mass and microarchitectural deterioration, is diagnosed most commonly in postmenopausal women and elderly men.(1) However, young or middle-aged men and premenopausal women can also suffer from osteoporosis. Most young individuals with osteoporosis have an underlying secondary cause, such as an endocrine or metabolic disorder (eg, hypogonadism) or medication exposure (eg, glucocorticoids).(2–11) However, 40% to 60% of young men and women with fractures and/or low areal bone mineral density (aBMD) by dual-energy X-ray absorptiometry (DXA) have no discernible secondary cause.(3,10,12–14) Such individuals are operationally considered to have idiopathic osteoporosis (IOP).

Measurement of aBMD by DXA is the current gold standard for diagnosing osteoporosis in postmenopausal women.(15) However, the clinical significance of low aBMD measurements in premenopausal women is uncertain, particularly in those who do not have a history of fragility fractures. Low aBMD measurements may be related to small bone size.(16,17) They may also be due to low peak bone mass, either genetically determined or related to suboptimal bone accrual during adolescence.(18) Under these circumstances, bone microarchitecture and strength may be normal and short-term fracture risk low. Conversely, low aBMD may be due to premature bone loss and associated with compromised bone microarchitecture and reduced bone strength. Newer technologies, capable of imaging the skeleton three-dimensionally with higher resolution than DXA, are required to determine whether there are specific microarchitectural abnormalities in premenopausal women with IOP.

Recently, an in vivo clinical imaging modality, high-resolution peripheral quantitative computed tomography (HR-pQCT), has been developed to assess bone microstructure (XtremeCT, Scanco Medical AG, Bassersdorf, Switzerland). The 3D data sets provided by HR-pQCT permit the separate analysis of cancellous and cortical bone at peripheral sites (distal tibia and radius).(19) The resolution of HR-pQCT allows for the visualization and quantitative evaluation of the internal structure of trabecular bone. Several clinical studies have demonstrated the ability of HR-pQCT to detect age- or disease-related changes in bone microarchitecture and to provide additional fracture risk determinants.(19–24) In addition, we recently applied HR-pQCT technology to a group of premenopausal women with IOP and detected abnormal trabecular microstructure and reduced mechanical competence.(25)

Despite its great potential, the current standard morphological analysis of HR-pQCT has several limitations. First, the resulting morphological parameters are based on a statistical average of the imaged trabecular network rather than on measurements of individual trabeculae. Second, the current analysis technique evaluates the morphology of trabecular bone at a global level without separate analyses for trabecular plates and rods, even though there are fundamental differences between these two types of structures. It is becoming increasingly apparent that the microstructural type of trabeculae (the two types of structures are plate-like and rod-like) is critical in determining the strength of trabecular bone(26–28) because a dramatic change in trabeculae from plate-like to rod-like occurs with aging and osteoporosis.(26–29) Furthermore, structure model index (SMI), a parameter used in micro–computed tomography (µCT) analysis to characterize the plate-likeness of the trabecular network,(30,31) is not included in the standard analysis output of HR-pQCT scans. Most importantly, several HR-pQCT standard parameters are derived rather than measured directly.(32) For example, the trabecular number (Tb.N*) parameter is defined as the inverse of the mean distance between the midline of trabeculae. Trabecular thickness (Tb.Th) and trabecular spacing (Tb.Sp) are derived based on Tb.N* and the derived bone volume fraction (BV/TV^d^) [ie, Tb.Th = BV/TV^d^/Tb.N* and Tb.Sp = (1 – BV/TV^d^)/Tb.N*] by analogy to standard histomorphometry.(33) Therefore, since these parameters are all highly dependent on BV/TV^d^, they may only provide limited additional information.

In our previous work we studied the relative importance of trabecular types (plates and rods) in the architecture and mechanical properties of trabecular bone and developed image-processing techniques for volumetrically segmenting the 3D trabecular bone microstructure as a collection of trabecular plates and rods.(27,28) We developed individual trabeculae segmentation (ITS)–based morphological analysis to assess trabecular microstructure based on measurements of each individual plate and rod.(27) One clinical application of ITS analysis was based on in vivo micro–magnetic resonance (µMR) images of tibial trabecular bone: ITS-based morphological analysis detected significant trabecular microarchitectural changes in plates and rods in response to testosterone treatment in hypogonadal men.(34) With the recent success of HR-pQCT in assessing microstructure and mechanical competence in osteoporosis, there is great potential in the application of ITS technology to clinical HR-pQCT images for in vivo and model-independent assessment of trabecular microarchitecture with a detailed quantification of trabecular types.

In this study, we applied ITS-based morphological analysis to HR-pQCT images of the distal radius and tibia to gain greater insight into trabecular bone microstructure changes in otherwise healthy premenopausal women with IOP. Then, the contributions of ITS-based microstructural parameters of HR-pQCT to mechanical properties estimated by micro–finite element (µFE) analysis of trabecular bone were evaluated. We hypothesized that trabecular microarchitecture in IOP would be characterized by conversion of trabecular plates into rods and decreased trabecular network connectivity and trabecular number, rather than uniform trabecular thinning.

## Materials and Methods

### Patient population

Premenopausal women (aged 20 to 48 years, 26 control and 31 IOP) were recruited at Columbia University and Creighton University. Subjects with IOP were included on the basis of a documented history of low-trauma fractures after age 18 and/or very low bone mineral density (BMD) measurement (*T*-score ≤ −2.5 or *Z*-score ≤ −2.0) at the lumbar spine, total hip, or femoral neck. Low-trauma fractures were defined as equivalent to a fall from a standing height or less, excluding skull or digit fractures. Fractures were ascertained by review of radiographs or radiograph reports. To qualify as normal controls, women were required to have normal aBMD by DXA (T-score ≥ −1.0) and no history of low-trauma fractures. Subjects were excluded if they had given birth or had lactated within the past 12 months. Patients and controls had a detailed history, physical examination, and biochemical evaluation to exclude secondary causes of osteoporosis, including disorders causing premenopausal estrogen deficiency, endocrinopathies (eg, hyperthyroidism, Cushing syndrome, prolactinoma), anorexia nervosa, bulemia, celiac or other gastrointestinal diseases, abnormal mineral metabolism (eg, osteomalacia, hyperparathyroidism), vitamin D deficiency, hypercalciuria (>300 mg/g of creatinine), and drug exposures (eg, glucocorticoids, anticonvulsants, anticoagulants, methotrexate).

### HR-pQCT images of the distal radius and distal tibia

HR-pQCT (XtremeCT, Scanco Medical AG) was performed at Columbia University for all participants, including those recruited at Creighton University. The nondominant forearm and distal tibia were immobilized in a carbon-fiber shell and scanned as described previously.(19,20,22) The region of interest was defined on a scout view by manual placement of a reference line at the endplate of the radius or tibia—the first slice being 9.5 and 22.5 mm proximal to the reference line at the radius and tibia, respectively. The HR-pQCT measurement included 110 slices corresponding to a 9.02-mm section along the axial direction with a nominal voxel size of 82 µm. The European forearm phantom was scanned whenever subjects were scanned for quality control.

**Fig. 1:**
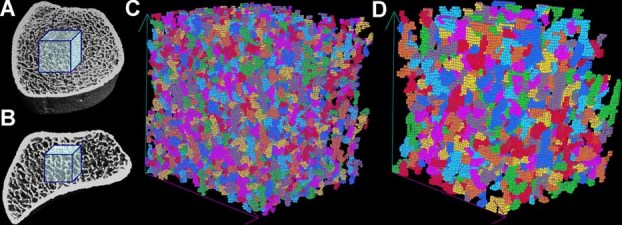
A 9.0 × 9.0 × 9.0 mm^3^ cubic subvolume of (*A*) distal tibia and a 5.7 × 5.7 × 5.7 mm^3^ subvolume of (*B*) distal radius were extracted and segmented into individual trabecular plates and rods (*C*, *D*). Colors were used to differentiate between individual trabeculae for display purposes only.

According to the standard patient evaluation protocol, the periosteal surfaces of the radius and tibia were contoured first semi-automatically and then by an automated threshold-based algorithm to separate the cortical and trabecular compartments.(32) The mineralized phase then was thresholded automatically using a Laplace-Hamming filter followed by a global threshold using a fixed value of 40% of the maximal grayscale value of the images.(35) These procedures were performed using Scanco software installed on an HP AlphaStation operating in a VMS environment (Hewlett-Packard, Palo Alto, CA, USA). After each image was thresholded, a 5.7 × 5.7 × 5.7 mm^3^ cubic subvolume corresponding to 70 × 70 × 70 voxels was extracted from the center of trabecular bone compartment of each image of the distal radius; similarly, a 9.0 × 9.0 × 9.0 mm^3^ subvolume corresponding to 110 × 110 × 110 voxels was extracted from each distal tibia (Fig. 1). Owing to the size difference of the distal radius and distal tibia segment, the cubic subvolume of the distal radius was smaller than that of the distal tibia.

### Individual trabeculae segmentation (ITS)–based morphological analysis of HR-pQCT images

All the trabecular bone subvolumes of the distal radius and distal tibia from the control and IOP subjects were subjected to ITS-based morphological analysis. First, a complete volumetric decomposition technique was applied to segment the trabecular network into individual plates and rods.(28) Briefly, digital topologic analysis (DTA)–based skeletonization(36) was applied first to transform a trabecular bone image into a representation composed of surfaces and curves skeleton while preserving the topology (ie, connectivity, tunnels, and cavities),(37,38) as well as the rod and plate morphology of the trabecular microarchitecture. Then digital topologic classification was applied in which each skeletal voxel was uniquely classified as either a surface or a curve type.(39) Using a newly developed iterative reconstruction method, each voxel of the original image was classified as belonging to either an individual plate or rod (Fig. 1). Based on the evaluations of dimension and orientation of each individual trabecular plate and rod, as well as junctions of surface and curve skeletons, a set of ITS-based morphological parameters was derived to quantify plate and rod bone volume fraction (pBV/TV and rBV/TV), axial bone volume fraction along the longitudinal axis (aBV/TV), plate and rod tissue fraction (pBV/BV and rBV/BV), that is, the volume of plate/rod bone tissue divided by the total volume of bone tissue, plate and rod number density (pTb.N and rTb.N, 1/mm), plate and rod thickness (pTb.Th and rTb.Th, mm), plate surface area (pTb.S, mm^2^), rod length (rTb.ℓ, mm), and rod-rod, plate-rod, and plate-plate junction density (R-R, P-R, and P-P Junc.D, 1/mm^3^). The definition of these ITS measurements can be found in the Glossary at the end of this article. Detailed methods of the complete volumetric decomposition technique and ITS-based measurements can be found in our recent publication.(27)

### Finite element analysis for HR-pQCT images

Each subvolume of HR-pQCT image of the distal radius and distal tibia was converted to a µFE model by directly converting bone voxels to 8-node elastic brick elements with an element size of 82 × 82 × 82 µm^3^. Using a customized element-by-element preconditioned conjugate gradient solver,(40) six µFE analyses were performed for each model, representing three uniaxial compression tests along three imaging axes (*x*, *y*, and *z*) and three uniaxial shear tests.(41) The general anisotropic stiffness matrix was first determined based on the results from the preceding analyses. A new coordinate system of orthotropic axes (*X*_1_, *X*_2_, and *X*_3_) representing the best orthotropic symmetry was then calculated using a numerical optimization algorithm, Powell's method,(42) to minimize an orthotropy objective function.(41) Transformation of the anisotropic matrix to a new coordinate system yielded the full orthotropic stiffness tensor.(41) The elastic constants and stiffness matrix were sorted such that *E*_11_ was in the direction of the lowest axial modulus and *E*_33_ was in the direction of the highest axial modulus (longitudinal axis). The elastic moduli (three Young's moduli, *E*_11_ < *E*_22_ < *E*_33_, and three shear moduli, *G*_23_, *G*_31_, and G_12_) were then derived from the orthotropic stiffness tensor. All the µFE analyses for subvolumes were implemented on a Dell XPS PC workstation (Dell, Inc., Round Rock, TX, USA).

### Statistical analysis

A nonparametric statistical test, a two-tailed Mann-Whitney *U* test, was performed to test for significant differences in the ITS measurements and the six elastic moduli between the control and IOP groups, as well as between the low BMD and fracture subjects in the IOP group. To investigate the contributions of the ITS measurements to the mechanical properties of trabecular bone, data from both the control and IOP groups were pooled. Each of the ITS measurements was correlated individually to the elastic moduli of trabecular bone at both the distal radius and distal tibia. Next, to differentiate the contributions of the ITS measurements to the mechanical properties of trabecular bone from that of BV/TV, partial correlation analyses between elastic moduli and ITS measurements with the effect of BV/TV removed were performed. A forward stepwise multiple linear regression was also performed to predict the elastic moduli by the ITS measurements. From the ITS-based parameters, pBV/TV, rBV/TV, aBV/TV, pBV/BV, pTb.N, rTb.N, pTb.Th, rTb.Th, pTb.S, rTb.ℓ, P-P Junc.D, P-R Junc.D, and R-R Junc.D were used as the independent variables. Finally, correlations between each of the ITS measurements were calculated for both the distal radius and tibia. All the statistical analyses were performed using KaleidaGraph 3.6 software (Synergy Software, Reading, PA, USA) or SPSS 13.0 software (SPSS, Inc., Chicago, IL, USA).

## Results

The study population consisted of 26 control individuals and 31 IOP subjects Twenty-one of these subjects were included because they experienced low-trauma adult fractures whether or not they had low aBMD (fracture group), and the other 10 had low aBMD but no adult low-trauma fractures (low BMD group). The mean ages of the control and IOP subjects were 34.3 ± 8.7 and 37.8 ± 7.8 years, respectively. The age, height, weight, and body mass index (BMI) did not differ between groups (*p* > .05). The mean *Z*-scores at the lumbar spine, total hip, femoral neck, and distal radius were 0.61 ± 0.82, 0.28 ± 0.63, 0.12 ± 0.75, and 0.71 ± 0.80 in the control group and −1.42 ± 1.16, −1.00 ± 1.30, −1.35 ± 1.22, and 0.18 ± 0.82 in the IOP group. Although most IOP subjects were included on the basis of low-trauma fractures regardless of aBMD, aBMD was lower at all sites in IOP subjects than in the control individuals (*p* < .05). Detailed characteristics of the study population were reported previously.(25)

**Fig. 2:**
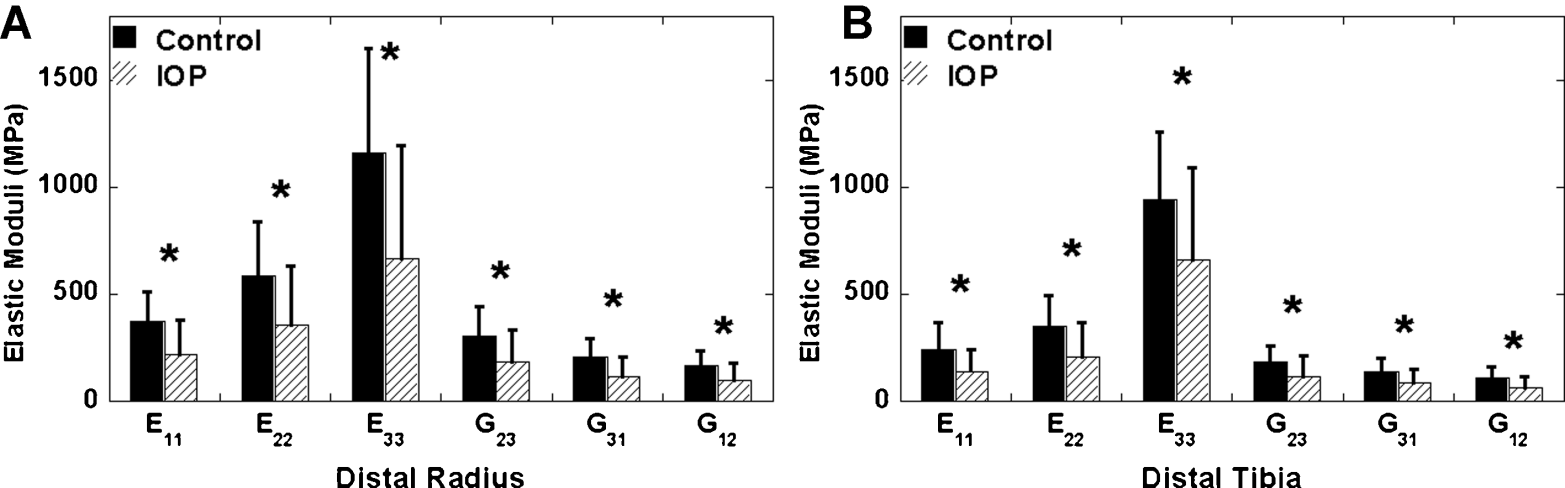
Comparisons of estimated elastic moduli between control and IOP groups at (*A*) the distal radius and (*B*) the distal tibia. The asterisk indicates a significant difference between control and IOP groups (two-tailed Mann-Whitney *U* test, *p* < .05).

The mean and standard deviation of the HR-pQCT image-based ITS and mechanical measurements of the control and IOP subjects are presented in Table 1 and Fig. 2. At the distal radius, plate bone volume fraction (pBV/TV), rod bone volume fraction (rBV/TV), and axial bone volume fraction (aBV/TV) were 42%, 23%, and 30% lower, and plate number (pTb.N) and rod number (rTb.N) were 20% and 10% lower in women with IOP. In addition, plate tissue fraction (pBV/BV) was 27% lower in women with IOP. In contrast, rod length (rTb.ℓ) was 10% higher and plate surface area (pTb.S) did not differ in IOP subjects. Rod-to-rod, plate-to-rod, and plate-to-plate junction densities (R-R, P-R, and P-P Junc.D) were 26%, 42%, and 45% lower in women with IOP. Similar results were found at the distal tibia. pBV/TV was 23% lower but not statistically significant (*p* = .1), and rBV/TV and aBV/TV were 31% and 18% lower in women with IOP. pTb.N and rTb.N were both 16% lower in women with IOP. However, pBV/BV did not differ. pTb.S and rTb.ℓ were 10% and 7% higher, and R-R, R-P, and P-P Junc.D values were 37%, 37%, and 34% lower in women with IOP. Neither pTb.Th nor rTb.Th differed in women with IOP at the distal radius or distal tibia (Table 1). At both the distal radius and the tibia, the six elastic moduli of the IOP group were significantly lower than those of the control group (Fig. 2). No statistical difference was detected in BV/TV, ITS measurements, or elastic moduli between fracture and low BMD subjects in the IOP group.

**Table 1:** ITS Measurements (Mean ± SD) of HR-pQCT Trabecular Bone Images at the Distal Radius and the Distal Tibia of Control and IOP Groups

	Distal radius			Distal tibia		
		
	Control (*n* = 26)	IOP (*n* = 31)	*p*	Control (*n* = 26)	IOP (*n* = 31)	*p*
pBV/TV	0.053 ± 0.026	0.031 ± 0.028	.0013	0.043 ± 0.019	0.033 ± 0.027	.0232
rBV/TV	0.171 ± 0.040	0.132 ± 0.039	.0002	0.147 ± 0.034	0.101 ± 0.037	.0001
aBV/TV	0.077 ± 0.025	0.054 ± 0.029	.0008	0.067 ± 0.015	0.055 ± 0.024	.0193
pBV/BV	0.22 ± 0.09	0.16 ± 0.09	.006	0.22 ± 0.08	0.23 ± 0.11	.94
rBV/BV	0.78 ± 0.09	0.84 ± 0.09	.006	0.78 ± 0.08	0.77 ± 0.11	.94
pTb.N (1/mm)	1.21 ± 0.25	0.97 ± 0.27	.0007	1.13 ± 0.15	0.96 ± 0.25	.0023
rTb.N (1/mm)	1.86 ± 0.22	1.67 ± 0.21	.0002	1.76 ± 0.19	1.48 ± 0.27	.0001
pTb.Th (mm)	0.192 ± 0.007	0.188 ± 0.011	.20	0.200 ± 0.008	0.202 ± 0.014	.82
rTb.Th (mm)	0.213 ± 0.007	0.213 ± 0.008	.76	0.215 ± 0.008	0.218 ± 0.008	.22
pTb.S (mm^2^)	0.138 ± 0.107	0.142 ± 0.152	.14	0.138 ± 0.130	0.153 ± 0.289	.02
rTb.ℓ (mm)	0.674 ± 0.048	0.734 ± 0.066	.0002	0.689 ± 0.038	0.736 ± 0.046	.0002
R-R Junc.D (1/mm^3^)	3.23 ± 0.90	2.39 ± 0.88	.0008	2.66 ± 0.93	1.67 ± 0.78	.0001
P-R Junc.D (1/mm^3^)	2.85 ± 1.10	1.64 ± 1.16	.0004	2.23 ± 0.74	1.40 ± 0.83	.0003
P-P Junc.D (1/mm^3^)	1.23 ± 0.54	0.67 ± 0.55	.0005	0.96 ± 0.36	0.63 ± 0.45	.0014

*Note: p* values were calculated by two-tailed Mann-Whitney *U* tests for comparison between groups. SD = standard deviation. Please refer to the Glossary for definitions of each ITS-based parameter.

At the distal radius, the ITS measurements, including pBV/TV, rBV/TV, aBV/TV, pBV/BV, pTb.N, rTb.N, pTb.Th, rTb.Th, and R-R, P-R, and P-P Junc.D, were significantly and positively correlated with BV/TV and the six elastic moduli of trabecular bone, whereas rBV/BV and rTb.ℓ were negatively correlated with BV/TV and the elastic moduli (*p* < .001; Table 2). pTb.S was weakly and negatively correlated with BV/TV but not statistically correlated with any elastic modulus (*p* > .05). The results from the distal tibia were similar to those from the distal radius with a few exceptions: rTb.Th had no contribution to any elastic modulus, and neither pTb.Th nor rTb.Th were correlated with BV/TV (Table 2). For both the distal radius and the distal tibia, the strongest correlations were between the axial modulus *E*_33_ and pBV/TV, aBV/TV, pTb.N, P-R Junc.D, and P-P Junc.D.

**Table 2:** Correlation Coefficient of Linear Regression and Partial Correlation Coefficient (Independent of BV/TV, Shown in Parentheses) of the ITS Measurements With the Elastic Moduli and BV/TV of HR-pQCT Images of Trabecular Bone at Both the Distal Radius and the Distal Tibia

	*E*_11_	*E*_22_	*E*_33_	*G*_23_	*G*_31_	*G*_12_	BV/TV
Distal radius							
pBV/TV	0.88* (0.48**)	0.93* (0.71*)	0.95* (0.81*)	0.95* (0.80*)	0.91* (0.64*)	0.90* (0.59*)	0.84*
rBV/TV	0.80* (−0.48**)	0.73* (−0.71*)	0.74* (−0.81*)	0.72* (−0.80*)	0.77* (−0.64*)	0.80* (−0.59*)	0.93*
aBV/TV	0.90* (0.34**)	0.93* (0.63*)	0.97* (0.80*)	0.96* (0.75*)	0.93* (0.53*)	0.92* (0.47*)	0.91*
pBV/BV	0.76* (0.33***)	0.83* (0.58*)	0.86* (0.70*)	0.86* (0.68*)	0.80* (0.50*)	0.79* (0.44*)	0.73*
rBV/BV	−0.76* (−0.33***)	−0.83* (−0.58*)	−0.86* (−0.70*)	−0.86* (−0.68*)	−0.80* (−0.50*)	−0.79* (−0.44*)	0.73*
pTb.N (1/mm)	0.90* (NS)	0.92* (0.45*)	0.94* (0.57*)	0.93* (0.54*)	0.92* (0.34**)	0.91* (NS)	0.95*
rTb.N (1/mm)	0.73* (−0.64*)	0.67* (−0.77*)	0.67* (−0.87*)	0.66* (−0.84*)	0.70* (−0.79*)	0.73* (−0.75*)	0.90*
pTb.Th (mm)	0.67* (NS)	0.65* (NS)	0.68* (0.27***)	0.66* (NS)	0.67* (NS)	0.67* (NS)	0.65*
rTb.Th (mm)	0.48* (0.38**)	0.54* (0.49*)	0.55* (0.53*)	0.55* (0.54*)	0.52* (0.47*)	0.50* (0.45*)	0.38**
pTb.S (mm^2^)	NS	NS	NS	NS	NS	NS	−0.27***
rTb.ℓ (mm^2^)	−0.83* (0.27***)	−0.81* (0.27***)	−0.82* (0.30***)	−0.81* (0.28***)	−0.83* (0.31***)	−0.84* (0.34***)	−0.94*
R-R Junc.D (1/mm^3^)	0.68* (−0.48*)	0.61* (−0.66*)	0.61* (−0.76*)	0.60* (−0.74*)	0.65* (−0.62*)	0.68* (−0.57*)	0.84*
P-R Junc.D (1/mm^3^)	0.93* (0.45*)	0.94* (0.61*)	0.96* (0.69*)	0.95* (0.68*)	0.95* (0.57*)	0.95* (0.51*)	0.96*
P-P Junc.D (1/mm^3^)	0.93* (0.50*)	0.95* (0.72*)	0.97* (0.83*)	0.97* (0.82*)	0.95* (0.67*)	0.94* (0.61*)	0.92*
Distal tibia							
pBV/TV	0.76* (0.37**)	0.84* (0.65*)	0.89* (0.79*)	0.89* (0.78*)	0.82* (0.59*)	0.82* (0.57*)	0.72*
rBV/TV	0.76* (−0.37**)	0.72* (−0.65*)	0.68* (−0.79*)	0.67* (−0.78*)	0.74* (−0.59*)	0.77* (−0.57*)	0.92*
aBV/TV	0.78* (NS)	0.82* (0.36**)	0.93* (0.81*)	0.84* (0.47*)	0.85* (0.46*)	0.78* (NS)	0.81*
pBV/BV	0.43* (0.42**)	0.49* (0.56*)	0.57* (0.74*)	0.56* (0.68*)	0.49* (0.58*)	0.47* (0.57*)	0.28***
rBV/BV	−0.43* (−0.42**)	−0.49* (−0.56*)	−0.57* (−0.74*)	−0.56* (−0.68*)	−0.49* (−0.58*)	−0.47* (−0.57*)	0.28***
pTb.N (1/mm)	0.83* (NS)	0.87* (0.37**)	0.92* (0.63*)	0.90* (0.51*)	0.87* (0.33***)	0.88* (0.33***)	0.89*
rTb.N (1/mm)	0.68* (−0.61*)	0.66* (−0.71*)	0.63* (−0.86*)	0.63* (−0.75*)	0.66* (−0.78*)	0.72* (−0.66*)	0.90*
pTb.Th (mm)	0.34*** (0.38**)	0.33*** (0.36**)	0.47* (0.67*)	0.37** (0.44*)	0.39** (0.52*)	0.31*** (0.35*)	NS
rTb.Th (mm)	NS	NS	NS	NS	NS	NS	NS
pTb.S (mm^2^)	NS	NS	NS	NS	NS	NS	−0.42**
rTb.ℓ (mm^2^)	−0.75* (NS)	−0.75* (NS)	−0.68* (0.43*)	−0.74* (NS)	−0.74* (NS)	−0.81* (NS)	−0.87*
R-R Junc.D (1/mm^3^)	0.63* (−0.36**)	0.59* (−0.56*)	0.50* (−0.86*)	0.54* (−0.66*)	0.60* (−0.57*)	0.66* (−0.42**)	0.81*
P-R Junc.D (1/mm^3^)	0.89* (0.33***)	0.93* (0.58*)	0.93* (0.55*)	0.94* (0.67*)	0.92* (0.47*)	0.94* (0.53*)	0.95*
P-P Junc.D (1/mm^3^)	0.87* (0.37**)	0.94* (0.69*)	0.94* (0.73*)	0.96* (0.82*)	0.91* (0.57*)	0.93* (0.61*)	0.89*

*Note*: Data were combined from both control and IOP groups.

* *p* < .001;

** *p* < .01;

*** *p* < .05.

NS: *p* > .05. Please refer to the Glossary for definitions of each ITS-based parameter.

Partial correlation analysis with the effect of BV/TV removed revealed that each ITS measurement contributed independently to the elastic moduli of trabecular bone. At the distal radius, pBV/TV, aBV/TV, pBV/BV, pTb.N, rTb.Th, rTb.ℓ, P-R Junc.D, and P-P Junc.D had significant and positive partial correlations with most elastic moduli of trabecular bone. rBV/TV, rBV/BV, rTb.N, and R-R Junc.D showed significant and negative partial correlations with most elastic moduli of trabecular bone. pTb.Th had positive and significant partial correlations with *E*_33_ but no correlation with the other moduli. At the distal tibia, pBV/TV, aBV/TV, pBV/BV, pTb.N, pTb.Th, P-R Junc.D, and P-P Junc.D had significant and positive partial correlations with most elastic moduli, whereas rBV/TV, rBV/BV, rTb.N, and R-R Junc.D had negative correlations. rTb.ℓ had significant and positive partial correlation with *E*_33_ but not with the other moduli (Table 2). Furthermore, multiple linear regression analysis revealed that each ITS-based microstructural parameter independently contributed to one or several elastic moduli of trabecular bone (Table 3).

**Table 3:** Correlation Coefficient and Independent Predictors of Multilinear Regression for the Predictions of Elastic Moduli of Trabecular Bone by the ITS Measurements

	Distal radius		Distal tibia	
		
Mechanical properties	Adjusted *r*	Independent predictors	Adjusted *r*	Independent predictors
*E*_11_	0.96	P-R Junc.D,* rTb.Th,* pTb.N,* rTb.ℓ,* pTb.Th***	0.93	P-R Junc.D,** rTb.Th,* R-R Junc.D,* pBV/BV***
*E*_22_	0.98	rTb.Th,* pBV/BV,* pBV/TV,* rTb.ℓ,* rTb.N***	0.95	P-P Junc.D,* rBV/TV,* rTb.N,** P-R Junc.D***
*E*_33_	0.99	P-P Junc.D,* rTb.Th,* aBV/TV,* pTb.N,* rTb.ℓ*	0.99	P-P Junc.D,* aBV/TV,* rTb.Th,* R-R Junc.D,* pTb.Th,* pTb.N,** pTb.S***
*G*_23_	0.99	P-P Junc.D,* rTb.Th,* pBV/BV,**** pBV/TV,*** rTb.ℓ,* pTb.N*	0.96	P-P Junc.D*
*G*_31_	0.98	P-P Junc.D,* rBV/TV,* rTb.N,* rTb.ℓ**	0.96	rTb.Th,* R-R Junc.D,* pBV/TV,* rTb.N**
*G*_12_	0.98	P-R Junc.D,*** rTb.Th,*** pTb.N,* rTb.ℓ,** pBV/TV,* rBV/TV,* rTb.N**	0.94	P-R Junc.D*

*Note*: The order of predictors is given in the order that predictors were entered in the forward stepwise regression model. The significance of each independent predictor is indicated by its *p* value (

* *p* < .001

** *p* < .01

*** *p* < .05

**** *p* < .1).

Please refer to the Glossary for definitions of each ITS-based parameter.

The correlations between ITS-based parameters are listed in Table 4. In general, plate-related parameters, including pBV/TV, pBV/BV, pTb.N, pTb.Th, P-R Junc.D, and P-P Junc.D, as well as aBV/TV, were significantly and positively correlated with each other at both the distal radius and the tibia. These parameters also significantly and negatively correlated with rBV/BV and rTb.ℓ.

**Table 4:** Correlation Coefficient of Linear Regression Between Each of the ITS Measurements of the HR-pQCT Images of Trabecular Bone at Both the Distal Radius and the Distal Tibia

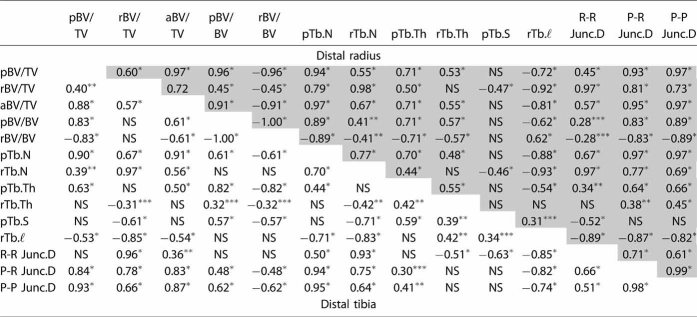

*Note*: Data were combined from both control and IOP groups. Data for the distal radius are highlighted in light gray.

*p < .001; **p < .01; ***p < .05. NS: *p* > .05. Please refer to the Glossary for definitions of each ITS-based parameter.

## Discussion

In this study, ITS-based morphologic analysis, which can provide model-independent and trabecular type-specific evaluations of trabecular bone microarchitecture, was applied to in vivo HR-pQCT images of the distal radius and tibia from premenopausal women with and without IOP. We found that premenopausal women with IOP had fewer longitudinally aligned trabeculae, fewer trabecular plates, fewer and longer trabecular rods, and decreased connectivity between rods and plates at both the distal radius and tibia. While both plate and rod bone volume fractions were significantly lower, trabecular plate and rod thickness did not differ in women with IOP at either the distal radius or distal tibia. These results suggest that there has been a loss of trabecular plates and rods in women with IOP, resulting in a less connected, less longitudinally aligned, and more widely separated trabecular network and reduced bone mass. Furthermore, the plate tissue fraction at the distal radius was significantly lower in women with IOP compared with controls, indicating trabecular conversion from plates to rods in the trabecular network. However, this change in plate tissue fraction was not found at the distal tibia. Within the IOP group, there was no statistical difference between fracture and low BMD subjects in any of the ITS-based parameters or elastic moduli. It is noteworthy to mention that the trabecular bone microarchitecture and mechanical properties in women with low BMD who had never had an adult low-trauma fracture were as severely affected as those who had had low-trauma fractures.

Previous retrospective transiliac biopsy studies of young adults with unexplained fractures have yielded conflicting data on bone microarchitecture.(11,43) In a recent report,(25) we examined in vivo microstructure measurements using the standard patient analysis protocol of HR-pQCT in the same populations of control and IOP subjects as the current study. Severe trabecular microarchitecture deterioration of the distal radius and distal tibia was detected in premenopausal women with IOP—reduced trabecular number and thickness and increased trabecular spacing and network inhomogeneity. The data reported in this study confirm the major findings we recently reported based on the standard patient analysis but also refine and extend them.(25)

High-resolution 3D images by HR-pQCT can reflect most trabecular microarchitecture when compared with gold standard µCT images(44); however, their potential is currently limited by the built-in microstructural analysis technique. Measurements such as Tb.Th and Tb.Sp are model-dependent. Furthermore, no measurements of trabecular type and trabecular network connectivity are provided by HR-pQCT standard analysis. In this study, we used a model-independent ITS technique to examine the differences in trabecular microstructure at the individual trabecula level. The ITS technique confirmed our previous findings using routine analysis of HR-pQCT scans, specifically that lower trabecular number and a more separated trabecular network underlie lower bone mass in premenopausal women with IOP. However, ITS-based analysis also provided additional detail about the trabecular structure in IOP, revealing a lower degree of trabecular alignment and loss of trabecular connectivity between trabecular rods and rods, rods and plates, and plates and plates in women with IOP. In contrast to standard HR-pQCT analysis measurements, which showed a significantly reduced Tb.Th, ITS analysis indicates that plate and rod thickness remained unchanged. There are two possible explanations for the contradiction. First, by HR-pQCT standard analysis, Tb.Th is a derived parameter, based on Tb.N*, calculated as the inverse of the distance between trabeculae midlines, and BV/TV^d^, a derived bone volume fraction assuming a density of fully mineralized bone tissue of 1200 mg HA/cm^3^. Therefore, the accuracy of the Tb.Th measurement by HR-pQCT standard analysis is subject to several assumptions. The variation may be even greater in pathologic conditions in which tissue density is altered. Second, however, compared with mean trabecular thickness (100 to 300 µm), HR-pQCT image voxel size (82 µm) may not be sufficient for ITS analysis to detect differences in trabecular thickness, especially if they are subtle.

The three Young's moduli and three shear moduli were all significantly lower in the IOP group than in the control group. Thus mechanical properties of trabecular bone in premenopausal women with IOP were severely compromised. Most ITS measurements were good indicators of the elastic moduli of trabecular bone. In general, linear correlations between the elastic moduli and ITS measurements suggested that pBV/TV, rBV/TV, aBV/TV, pBV/BV, pTb.N, rTb.N, pTb.Th, R-R Junc.D, P-R Junc.D, and P-P Junc.D correlate positively, whereas rBV/BV and rTb.ℓ correlate negatively with the mechanical properties of trabecular bone. Although most ITS measurements were also significantly associated with BV/TV (Table 2), partial correlation results showed that at a fixed BV/TV, plate-associated parameters such as pBV/TV, aBV/TV, pBV/BV, pTb.N, pTb.Th, P-R Junc.D, and P-P Junc.D contribute positively to the mechanical properties of trabecular bone at both the distal radius and the distal tibia. In contrast, rod-associated parameters such as rBV/TV, rBV/BV, rTb.N, and R-R Junc.D correlate negatively with trabecular bone's mechanical properties. This is consistent with our findings based on µCT images of human trabecular bone that trabecular plates play an important role in determining the mechanical properties of bone and that a conversion of trabecular network from plates to rods impairs bone strength.(27,28)

There were also interdependencies between most of the ITS measurements (Table 4). For example, an increase of pBV/TV is associated with an increase of pBV/BV, pTb.N, P-P Junc.D, pTb.Th, and aBV/TV, as well as a decrease of rBV/BV and rTb.ℓ. However, although associated with each other, each ITS measurement independently contributes to one or more elastic moduli, as demonstrated by multiple linear regressions of the elastic moduli with ITS-based parameters (Table 3). The combination of ITS measurements showed excellent predictions of the mechanical properties of trabecular bone, with correlation coefficient *r* ranging from 0.96 to 0.99 at the distal radius and 0.93 to 0.99 at the distal tibia.

In this study, the elastic moduli of trabecular bone were estimated by HR-pQCT image-based µFE analysis based on a subvolume of trabecular region at the distal radius and the distal tibia. This µFE analysis technique has been thoroughly validated on HR-pQCT images with the registered µCT images as a gold standard.(44) Correlations between HR-pQCT- and µCT-based elastic moduli of trabecular subvolumes were highly significant, with *r*^2^ > 0.90.(44) The accuracy of ITS measurements was also investigated in a preliminary validation study.(45) Most ITS measurements of HR-pQCT images correlated significantly with those of the registered µCT images, and plate-related parameters yielded better correlations than rod-related parameters. This suggests that the partial-volume effect in the low-resolution regimen has a greater influence on trabecular rods than plates. However, junction densities (P-P, P-R, and R-R Junc.D), determined by counting junctions of 1-voxel-thick trabecular structure, did not correlate between the two modalities. Therefore, conclusions regarding these parameters should be interpreted cautiously. Interestingly, however, Junc.D of HR-pQCT significantly contributed to the estimated elastic moduli of trabecular bone. Furthermore, they were highly discriminative in detecting differences between premenopausal women with and without IOP.

The current HR-pQCT and ITS-based morphologic analysis has several limitations. A global threshold technique provided by the HR-pQCT manufacturer was used to obtain the input image for ITS-based analysis. This threshold technique has the tendency to overestimate trabecular bone volume, number, and thickness measured by model-independent methods.(44) In addition, the image voxel size, 82 µm, is close to the low end of trabecular thickness. A subvoxel process(46) followed by a local adaptive threshold technique(47) would be expected to improve the voxel-based microstructural and mechanical measurements of images in the low-resolution regime. In future studies it will be of interest to apply these techniques to in vivo HR-pQCT images to improve voxel-based analysis. We expect that these preprocessing techniques will also improve the correlations of the ITS measurements of HR-pQCT with their gold standard µCT measurements.

In conclusion, ITS-based morphological analysis of HR-pQCT images detected significant reductions in trabecular plate and rod number and bone mass, leading to a less connected, less longitudinally aligned, and more widely separated trabecular network and reduced bone mass at the distal radius and distal tibia in premenopausal women with unexplained osteoporosis. On the other hand, trabecular plate and rod thickness remained unchanged. A transition to a more rod-like trabecular microstructure was found in the distal radius but not in the distal tibia. Most ITS measurements contributed significantly to the elastic moduli of trabecular bone over and above the bone volume fraction. This study provides in vivo evidence that trabecular plates play a far more important role than rods in determining the elastic moduli of trabecular bone. At a fixed bone volume fraction, platelike trabeculae contribute positively to the mechanical properties of trabecular bone. These findings suggest that ITS-based morphological analysis of HR-pQCT images provides additional information regarding bone strength and is a sensitive and promising clinical tool for the investigation of trabecular bone microstructure.

## Glossary

Definition of the ITS-Based Microstructural Parameters

pBV/TV: Plate bone volume fraction: the total volume of trabecular plates divided by the bulk volume
rBV/TV: Rod bone volume fraction: the total volume of trabecular rods divided by the bulk volume
aBV/TV: Axial bone volume fraction: the total volume of trabeculae aligned with the longitudinal axis divided by the bulk volume
pBV/BV: Plate tissue fraction: the total volume of trabecular plates divided by the total bone volume
rBV/BV: Rod tissue fraction: the total volume of trabecular rods divided by the total bone volume
pTb.N (1/mm): Trabecular plate number density: the cubic root of the total number of trabecular plates divided by the bulk volume
rTb.N (1/mm): Trabecular rod number density: the cubic root of the total number of trabecular rods divided by the bulk volume
pTb.Th (mm): Mean trabecular plate thickness: the average thickness of trabecular plates
rTb.Th (mm): Mean trabecular rod thickness: the average diameter of trabecular rods
pTb.S (mm^2^): Mean trabecular plate surface area: the average surface area of trabecular plates
rTb.ℓ (mm): Mean trabecular rod length: the average length of trabecular rods
R-R Junc.D (1/mm^3^): Rod-rod junction density: the total number of junctions between rod and rod divided by the bulk volume
P-R Junc.D (1/mm^3^): Plate-rod junction density: the total number of junctions between plate and rod divided by the bulk volume
P-P Junc.D (1/mm^3^): Plate-plate junction density: the total number of junctions between plate and plate divided by the bulk volume
